# Improving the Long-Range Intramolecular Proton Transfer—Further Molecular Design of the Successful Molecular Switch 8-(Benzo[d]thiazol-2-yl)quinolin-7-ol (HQBT)

**DOI:** 10.3390/molecules30091935

**Published:** 2025-04-26

**Authors:** Daniela Nedeltcheva-Antonova, Liudmil Antonov

**Affiliations:** 1 Institute of Electronics, Bulgarian Academy of Sciences, 1784 Sofia, Bulgaria; 2 Institute of Organic Chemistry with Centre of Phytochemistry, Bulgarian Academy of Sciences, 1113 Sofia, Bulgaria

**Keywords:** molecular switch, proton crane, tautomerism, proton transfer, benzothiazole, 7-hydroxy quinoline, DFT

## Abstract

Previously, we have described a successful molecular switch (8-(benzo[d]thiazol-2-yl)quinolin-7-ol), working on the basis of long-range proton transfer. Bearing in mind that its switching efficiency in low-polarity aprotic solvents is not sufficient, in the current communication, we investigate in detail the effect of the substitution in the benzothiazole ring. By using the DFT approach, the ground-state stability of the tautomeric forms, involved in the switching process, is modeled with the aim of finding conditions where clean switching could be possible in variety of aprotic solvents. The results indicate that the substitution with electron-acceptor substituents could increase the switching efficiency, but the overall improvement depends on the positions and electronic effect of the particular substituent.

## 1. Introduction

Hydrogen bonding is one of the most intriguing phenomena in organic chemistry [1,2,3], with vital importance in biology [4,5,6], drug design [7,8] and in various fields of materials science [9,10,11,12]. In many of the cases, the practical applicability of the hydrogen bonding is related to the proton transfer (PT) [13] that occurs between the proton donor and proton acceptor parts involved in this interaction. The short-range intramolecular proton transfer that occurs directly as a result of hydrogen bonding either in the ground (GSIPT) or in the excited (ESIPT) state [13,14] is a well-studied process with many implications in light-emitting materials and laser dyes [15,16,17,18,19,20,21,22], optical sensors [23,24,25], organic light-emitting diodes [26,27,28,29,30,31,32,33,34,35] and photo stabilizers [36]. The long-range intramolecular PT, where the proton donor and proton acceptor parts of the molecule are separated in a way that makes hydrogen bonding impossible, is much more intriguing. Whether it happens either through the assistance of other molecules, which transport the proton, or truly intramolecularly, this process is a complex phenomenon which includes, as a rule, short-range PTs as elementary steps.

7-Hydroxyquinoline is a typical example, where long-range proton transfer occurs under suitable conditions [37,38,39,40,41,42,43,44,45,46,47]. The acidic OH group and the basic N atom in the ring are at a distance that does not allow an intramolecular hydrogen bond to form. Thus, the keto tautomer has been experimentally observed only either in protic organic solvents or in the presence of water and results from the intermolecular, solvent-assisted PT mechanism, confirmed either experimentally [47,48] or theoretically [49,50,51,52,53,54,55,56,57]. In aprotic non-polar solvents, the proton exchange results from formation of cyclic dimers [58,59,60,61,62]. Although the overall effect leads to proton transport within the molecule, the solvent or concentration-assisted mechanism gives a good reason to classify this process as pseudo-intramolecular [63].

**Scheme 1:**
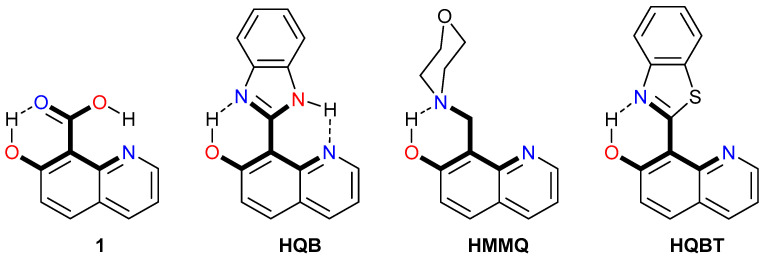
Molecules, where long-range intramolecular PT occurs. The proton exchange skeleton is given in bold. The abbreviations of the compounds used in the original papers are shown.

Under suitable structural conditions (Scheme 1), a proton can be transferred truly intramolecularly. However, there are some details to be considered in addition. Double PT occurs upon excitation in **1 [64]** and **HQB [65]**. The release of the OH proton to the carboxylic/imidazole moiety initiates delivery of the carboxylic/imidazole proton to the quinoline N atom and the delivered proton is not the original one. Historically, the first system **HMMQ**, where the original proton is delivered, was developed by Varma and co-workers [66,67] and includes a morpholine rotor attached to the tautomeric stator by a single-bond axle. Very recently, we have described a tautomeric molecular switch **HQBT**, where the proton is delivered to the quinolyl nitrogen atom with high efficiency [68]. In both cases (**HMMQ** and **HQBT**), upon photoexcitation, as a first step, the OH proton is picked up by the nitrogen atom from the morpholine/benzothiazole part (short-range PT) via the ESIPT process and then through a series of steps, different in **HMMQ** and **HQBT** [68,69,70], is delivered to the quinolyl nitrogen like a cargo.

**Scheme 2:**
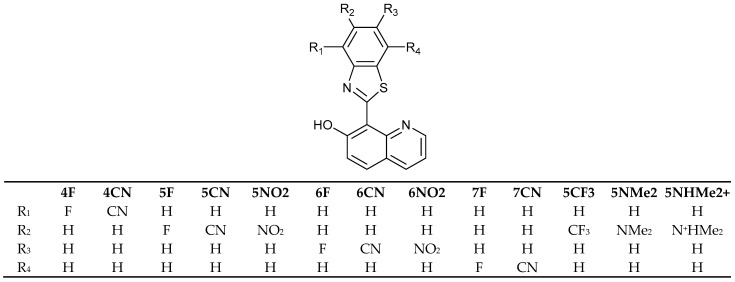
Compounds discussed in current investigation.

Following the detailed investigation of the mechanism of action of **HQBT** [68], structural modifications have been modeled to improve its performance by replacing the benzothiazole rotor by other heterocycles [71,72,73,74,75,76] or by using an external electric field as a trigger of switching [77]. No perfect solution has been found up to now. Therefore, in the current study we will discuss the molecular design based on various substitutions in the benzothiazole part of **HQBT**, as shown in Scheme 2. For this purpose, we will use the DFT/TDDFT protocol developed and validated by the experimental measurements and post-HF methods in [68]. The obtained theoretical results will be considered in the light of an overall discussion about the efficiency of switching in this class of compounds. As seen, a selection of electron-acceptor substituents with various electronic effects (F and CN) are considered on all possible positions, while the bulky substituents (NMe_2_, NO_2_ and CF_3_) are placed in a way (positions 5′ and 6′) that avoids either steric hindrance or the formation of additional intramolecular hydrogen bonding, which could affect the switching.

## 2. Results and Discussion

**Figure 1:**
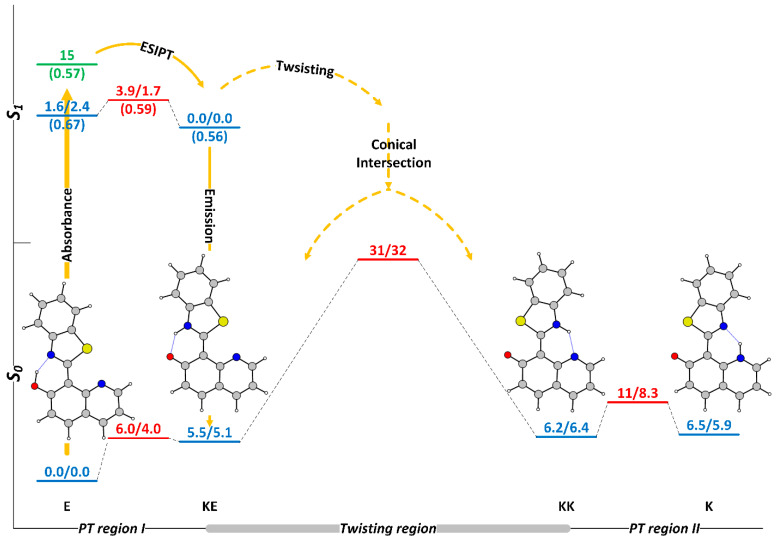
Sketch of the potential energy surface (PES) of **HQBT** in toluene in the ground (M06-2X/TZVP) and in the first singlet excited (CAM-B3LYP/TZVP) state. The stationary tautomeric forms are indicated in blue, the transition states—in red, the Frank-Condon state—in green. All tautomeric forms have been found to be strictly planar. The numerical values present relative energy ΔE/relative Gibbs free energy ΔG and are given in kcal/mol units. Both PESs are scaled against the relative energies. Excited-state oscillator strength is given in in brackets. ΔE and ΔG values in the first singlet excited state are calculated in respect to KE*.

The experimentally and theoretically confirmed switching process in **HQBT** [68,76] is sketched in Figure 1 and can be summarized as follows. The **E** tautomer that exists only in the ground state (denoted here as off-state of switching), upon photoexcitation undergoes very fast ESIPT, giving KE* (here and below * indicates first singled excited state), which either returns back to the ground-state emitting and then through GSIPT restores the initial enol, or through twisting goes to the conical intersection (CI) region, populating simultaneously both intermediate keto tautomers **KE** and **KK**. The obtained **KK** form can either return back to **E**, overcoming the twisting barrier (**TS(KE-KK)**), or can through N to N GSIPT give rise to **K**, the desired end keto tautomer (denoted as on-state of switching), which then relaxes thermally to **E**. The splitting of KE* into two directions defines two photocycles to govern the switching process—the very fast **E**-KE*-**KE**-**E** and the relatively slow **E**-KE*-**KK**-**K**-**E**. Both processes are experimentally confirmed, first by the appearance of ESIPT emission and secondly by the rise and then disappearance of red-shifted absorption.

This simplified description of the switching immediately indicates two key parameters in respect to the efficiency: the relative stability of **KK** and **K**, and the twisting barrier. If the energy of **KK** is lower than **K**, the latter cannot be populated and the switching is not completed, as happens in the Schiff bases, derived from 7-hydroxy quinoline [78]. As seen from Figure 1, in toluene, the relative energies of **KK** and **K** are almost equal, which in practical terms leads to partial switching, while in acetonitrile, where the end keto tautomer is more stable, the switching is almost complete [68].

The twisting barrier in respect of **KK** (ΔE(**TS(KE-KK)**-**KK**) or ΔG(**TS(KE-KK)**-**KK**)) along with the GSIPT barrier **TS(KK-K)** determines which part of the **KK** molecules obtained as a result of the deactivation of KE* through CI would go into the needed direction of **K**. In a perfect condition (assuming that there is no tunnelling or triplet state involvement), the twisting barrier determines the life-time of the on-state and a large barrier is desired if long-lived switches are aimed for. The relatively unstable **KK** in respect of this barrier is the major reason for very fast backward thermal relaxation in 2-(2′-hydroxyphenyl)benzoxazole [79] and 2-(2′-hydroxyphenyl)benzothiazole [80,81,82].

The overall discussion up to now allows to define some conditions needed to provide efficient switching, based on long-range intramolecular PT.

1. The off-state (**E**) should be only present initially, which defines that the energy stabilization in respect to the rest of the tautomers (**KE**,**KK**,**K**) should be substantial to prevent their appearance under the equilibrium conditions.
2. The on-state **K** should be substantially more stable than **KK** in order to assume full transition from the latter to the former.
3. The twisting barrier in respect of **KK** should be large enough to assure predominant and efficient GSIPT to **K**.
4. The GSIPT barriers (**TS(E-KE)** and **TS(KK-K)**) should be as low as possible to prevent **KE** and **KK** from being trapped.
5. The ESIPT process should lead to simultaneous twisting in order to get predominantly twisted instead of a planar KE*.

As seen, these conditions are generally defined and could be considered not as exact absolute values, but as relative parameters. Usually for conditions 1 and 2, values of larger than 2–3 kcal/mol are considered as sufficient, because the switching is monitored by optical spectroscopy and traces of the undesired forms below 2–3% are hard to detect.

Returning back to **HQBT**, it is seen that in all studied solvents, condition 1 is fulfilled, while condition 2 is a result of the used solvent [68]. The on-state is substantially more polar, which leads to its stabilization in polar solvents, and as a result the switching is much more efficient in acetonitrile. Conditions 3 and 4 also depend on the solvent, but the overall result is that the life-time of **K** is in the seconds timescale [68], which allows the switching process to be monitored by conventional optical spectroscopy. Condition 5 will be discussed in detail later. Following the overall discussion up to now, the possible directions for improvement of **HQBT** could be to provide more efficient switching in non-polar solvents (condition 2) and to make the on-state long-living by tuning the twisting barrier.

In Table 1, the natural charges of the proton donor and proton acceptors in **HQBT** are collected. It is seen that the basicity (in broad terms) of the quinolyl nitrogen atom is lower comparing to the thiazolyl one, which fits the lower stability of the **K** tautomer. Consequently, the reduction in the electron density at the latter by adding electron-acceptor substituents at the benzothiazolyl rotor could lead to situation that the above-discussed condition 2 could be fulfilled in low-polarity solvents. According to our previous study [76], the rotor–stator system in **HQBT** has a well-defined donor–acceptor charge-transfer character in **E** and **K** and acceptor–donor in the intermediate keto tautomers. In this way, the implementation of electron-acceptor groups in the benzothiazole unit should increase the conjugation between the rotor and the stator in **KE** and **KK** and, hence, increase the twisting barrier. These assumptions explain the structures to be modelled, as shown in Scheme 2.

The basic information about the ground-state PES in toluene of the compounds from Scheme 2 is collected in Table 2 and Table S1. PESs of each compound can be seen in Figure S1. As seen from the data in the tables, in all studied compounds the enol tautomer is substantially more stable and the appearance of the keto tautomers could be excluded. In all compounds, the GSIPT from **KE** to **E** is predicted to be barrierless. In all electron-acceptor substituents, depending on the type and position, the **KE** and **KK** are destabilized in respect to **HQBT**, and the twisting barriers are getting higher. The opposite is seen for **4NMe2**, taken as a counter example.

**Figure 2:**
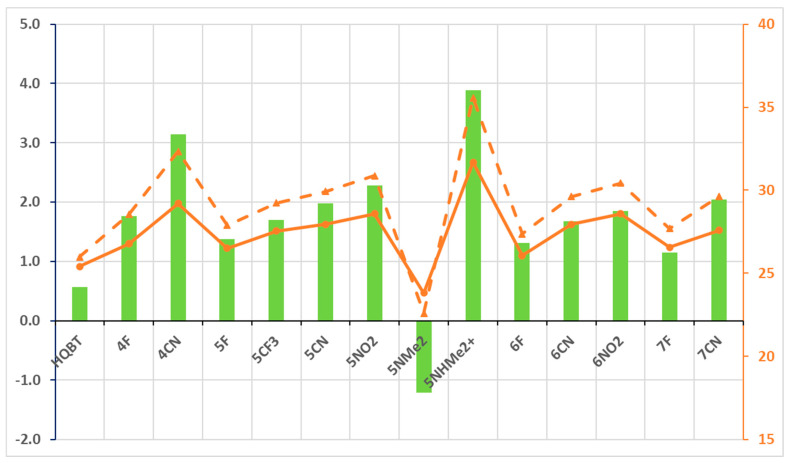
Parameters related to condition 2: ΔG(**KK**-**K**) in green bars (against left axis) and barriers of backward-twisting to **E**—ΔG(**TS(KK-K)**-**K**), in orange dashes, and ΔG(**TS(KK-K)**-**KK**), in orange solid line, in both—(against the right axis). All values (M06-2X/TZVP) are in kcal/mol units.

Bearing in mind that both **KK** and **TS(KK-K)** increase in energy upon electron-acceptor substitution, more detailed analysis is needed in respect of the aimed-for properties. Figure 2 shows the difference in relative stabilities of **KK** and **K** along with the height of the twisting barrier. Taking **HQBT** as a base for comparison, the implementation of electron-acceptor substituents leads to stabilization of **K** in respect to **KK**, providing conditions for clean switching from **E** to **K**. It is obvious that in most of the cases (except for some F substituents), the backward process of relaxation from **K** to **E** is expected to be substantially slower. Here, **5NHMe2+** could be considered as a border limit of decrease in basicity of the thiazolyl nitrogen atom (which leads to disappearance of **KE**) and increased rotor–stator conjugation in the intermediate keto forms, both caused by a single functional group. It should be noted that **5NHMe2+** is a computational chimera, because it is impossible to achieve this effect in practice by simple protonation of **5NMe2**, where the quinolyl and thiazolyl nitrogen atoms have substantially higher electron densities and would be preferred as protonation sites. The figure illustrates the opposite direction of action in **5NMe2**, where the implementation of the electron-donating substituent leads to stabilization of **KK** and relative destabilization of the **K** form in such a way that it cannot be populated.

**Figure 3:**
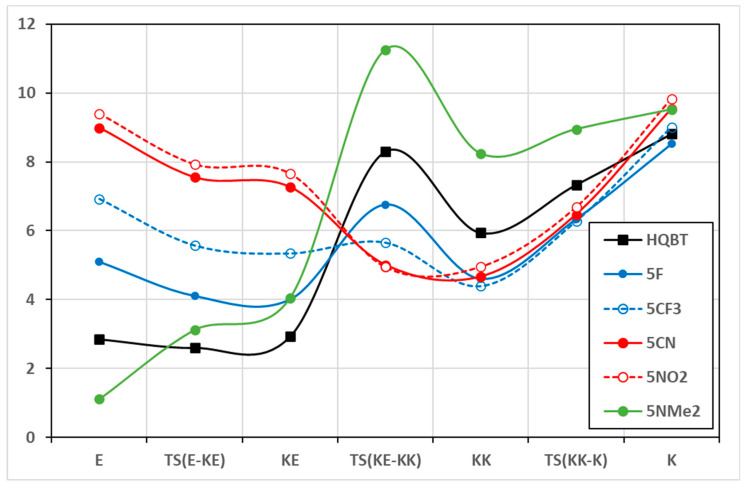
Dipole moments (in Debay units) of the tautomers and the transition states of selected compounds in toluene. Detailed information is available in Table S2.

The change of the solvent environment is the other factor that strongly affects the PES and the switching efficiency. In the case of **HQBT**, as already shown, the transfer from toluene to acetonitrile leads to stabilization of **K** in respect of **KK** and **E** and reduction in the twisting barrier. These changes are in substantial part a result of the implicit solvation effect on the dipole moments of the tautomers and transition states involved in the long-range proton transfer. The dipole moments are collected in Table S2 and presented in Figure 3 and Figures S2 and S3. As seen in the case of **HQBT**, the **K** tautomer and the twisting transition state are the most polar, and the increase in the solvent polarity leads to their additional stabilization. In this particular case, the stabilization of **K** in respect of **KK** is positive, leading to complete switching, while the reduction in the twisting barrier makes the on-state less long-living. The effect of the change of position of the electron-acceptor group from position 4′ to 7′ leads to a decrease in the dipole moments of the pair **E**/**KE** and an increase in these of **KK**/**K**, which makes the stabilization of **K** in more polar solvents expected. The effect, as seen in Figure S2, is more pronounced in the case of the CN substituent compared to F. As seen in Figure 3 and Figure S3, in the case of 5′ and 6′ substitution, the dipole moments of the on- and off-states become larger, and the polarity of the twisting transition state becomes lower. This is a highly positive effect, more pronounced in the CN and NO_2_ substituents, and leading to stabilization of the needed tautomers (**E** and **K**) without much effect on the lifetime of the switched form.

**Figure 4:**
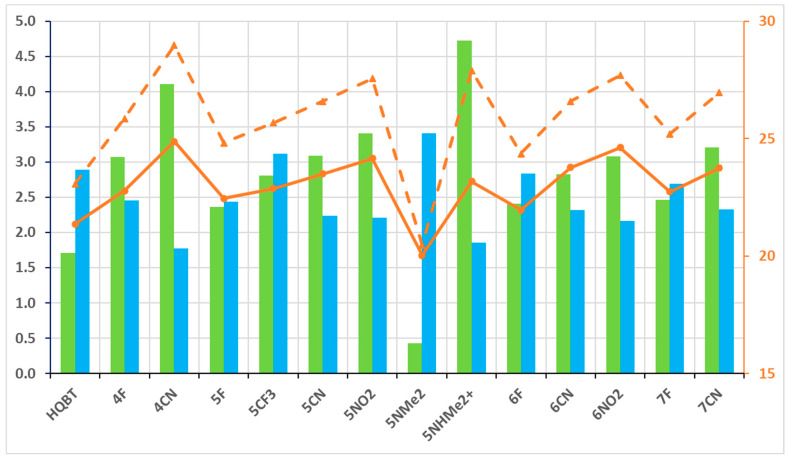
Parameters related to condition 2: ΔG(**KK**-**K**) in green bars (against left axis) and barriers of backward-twisting to **E**—ΔG(**TS(KK-K)**-**K**), in orange dashes, and ΔG(**TS(KK-K)**-**KK**), in orange solid line, in both—(against the right axis). The value of ΔG(**K-E**), related to condition 1, is given in blue bars, against the left axis. All values (M06-2X/TZVP) are in kcal/mol units.

The relative energies of the ground-state tautomers in acetonitrile are collected in Tables S3 and S4. The most important information is summarized in Figure 4, showing that the transition to the more polar aprotic solvent leads to substantial stabilization of **K** in respect to **KK**, which is a needed condition for efficient switching. As seen, the best performers are the cyano and nitro substituents on the 5′ and 6′ positions in the benzothiazole ring. The **4CN** also performs well, but the **K** tautomer is too stabilized, and some traces of it could be expected in the equilibrium state, which places the clean switching under question (condition 1 above). In general, taking all information into account, from toluene and acetonitrile, **4CN** and **5NO2** could be good candidates to be studied experimentally. In analogy to **HQBT** [68], these compounds give a good possibility for experimental detection of the switching process. As seen from Figure S4, the absorption spectrum of the on-state (**K** tautomer) is intensive and is red-shifted in respect to the E form in both cases, but the changes are more pronounced for **5NO2**.

As it has been discussed above, the ESIPT is the first step in the switching process. Ideally, the excited-state proton transfer should be accompanied by simultaneous twisting of the rotor. In such a case, the emission channel from KE* would be closed and all excited molecules could go to the CI region, increasing the overall switching efficiency. Previously, it was predicted that such behavior is possible in the pyridine rotors [76]. In the particular case here, the compounds from Scheme 2 keep strict planarity in the excited **E** and **KE** forms. In order to see how the substitution affects the ESIPT, we compare **HQBT** with **5NO2** and **5NMe2**. The relative energies are collected in Table 3.

As seen from Table 3 and confirmed experimentally [68], the ESIPT in **HQBT** is barrierless in both solvents. Compared to it, the substitution in the benzothiazole ring hampers the process in the case of the NMe_2_ group. In the case of **5NO2**, no substantial improvement could be expected, and the process remains barrierless with parameters comparable to **HQBT**. This means that, in the excited state, **5NO2** is expected to act in a manner similar to **HQBT**.

## 3. Theoretical Methodology

Quantum-chemical calculations were performed using the Gaussian 16 C.01 program suite [83]. All structures (in both ground and excited states) were optimized without restrictions in the corresponding solvent environment, using tight optimization criteria and an ultrafine grid in the computation of two-electron integrals and their derivatives. The true minima were verified by performing frequency calculations in the corresponding solvent environment. The used implicit solvation was described by the Polarizable Continuum Model [84] (the integral equation formalism variant, IEFPCM, as implemented in Gaussian 16). The transition states were estimated using the transit-guided quasi-Newton (STQN) method [85] and again verified by performing frequency calculations in the corresponding environment. Natural Bond Orbital analysis was performed using NBO version 3 [86], as implemented in Gaussian 16.

The M06-2X [87,88] functional with the TZVP [89] basis set was used for the structure optimizations in the ground state. The use of M06-2X provides very good predictability in the ground-state [68,78,90,91,92,93] tautomeric composition in tautomeric compounds and proton cranes in solution as well as the E/Z isomerization ratio in some rotary switches.

The TD-DFT method [94,95,96] was used for the singlet excited-state optimizations. CAM-B3LYP [97] with the TZVP basis set was used for the optimizations. The selection of CAM-B3LYP is based on its better performance (in comparison with variety of density functionals, including M06-2X) in describing electronic excitation energies, excited-state geometries, dipole moments and oscillator strengths in a variety of systems [98,99,100,101], including ESIPT ones [102], as well as in our own previous experience [68,78,103]. Previously, we have shown [68] that, in the case of **HQBT**, the results for the excited-state PESs, obtained by M06-2X [87,88] and CAM-B3LYP, are essentially the same.

In addition, the DFT results for both ground and excited states in **HQBT** were previously validated [68] by using domain-based local pair natural orbital coupled-cluster singles and doubles and perturbative triple excitations (DLPNO-CCSD(T) [104]) and domain-based local pair natural orbital-similarity-transformed equation-of-motion coupled-cluster singles and doubles (DLPNO-STEOM-CCSD [105]), respectively.

The UV-Vis spectra were predicted by the B3LYP [106] functional (6-311+G(2d,p) basis set) using the M06-2X optimized ground-state geometries. The spectra were simulated according to the methodology described previously by us [107] by using a single Gaussian band shape with a half-band width (${\Delta \nu }_{1/2}$) of 3000 cm^−1^.

## 4. Conclusions

A series of compounds, derived from **HQBT**, are considered theoretically in respect to the efficiency of the long-range intramolecular PT-based switching. The results indicate that the implementation of electron-acceptor substituents in the benzothiazole ring leads to improved switching efficiency depending on the nature and position of the substituent. Taking all obtained information into account, from toluene and acetonitrile, **4CN** and **5NO2** are promising candidates to be studied experimentally.

## Figures and Tables

**Table 1:** Natural charges of the oxygen and nitrogen atoms of the tautomers of **HQBT** in toluene (M06-2X/TZVP).

Tautomer	O	N_thiazolyl_	N_quinolyl_
**E**	−0.662	−0.505	−0.444
**KE**	−0.685	−0.511	−0.461
**KK**	−0.642	−0.522	−0.504
**K**	−0.634	−0.532	−0.479

**Table 2:** Tautomeric and transition states presented as relative Gibbs free energies (in kcal/mol units) in toluene (M06-2X/TZVP).

	E	TS(E-KE)	KE	TS(KE-KK)	KK	TS(KK-K)	K
**HQBT**	0.00	3.97	5.08	31.85	6.44	8.26	5.87
**4F**	0.00	4.90	6.12	33.53	6.76	7.79	4.99
**4CN**	0.00	5.70	6.78	35.89	6.69	7.21	3.55
**5F**	0.00	4.33	5.56	33.21	6.70	8.17	5.32
**5CF3**	0.00	4.56	5.81	34.87	7.32	8.73	5.63
**5CN**	0.00	4.95	6.03	35.04	7.10	8.28	5.12
**5NO2**	0.00	5.19	6.28	35.71	7.12	8.22	4.84
**5NMe2**	0.00	3.19	4.48	29.58	5.78	8.12	6.99
**5NHMe2+**	0.00	-	-	40.90	9.23	9.77	5.34
**6F**	0.00	4.51	5.74	33.17	7.11	8.67	5.79
**6CN**	0.00	4.74	5.85	35.07	7.12	8.38	5.44
**6NO2**	0.00	4.79	5.96	35.74	7.16	8.34	5.32
**7F**	0.00	4.45	5.67	33.67	7.12	8.51	5.97
**7CN**	0.00	4.96	6.13	35.39	7.80	9.08	5.76

**Table 3:** Relative energies (CAM-B3LYP/TZVP, in kcal/mol units) in toluene and in acetonitrile (in brackets) of **HQBT**, **5NO2** and **5NMe2**.

	5NMe2		HQBT		5NO2	
	ΔE	ΔG	ΔE	ΔG	ΔE	ΔG
E*	0.0 (1.45)	0.55 (1.93)	1.62 (2.82)	2.35 (3.23)	1.26 (2.42)	2.35 (2.81)
TS(E*-KE*)	3.80 (4.30)	1.20 (2.12)	3.87 (4.84)	1.74 (2.67)	3.62 (4.58)	1.44 (2.29)
KE*	0.52 (0.0)	0.0 (0.0)	0.0 (0.0)	0.0 (0.0)	0.0 (0.0)	0.0 (0.0)

## Data Availability

The original contributions presented in this study are included in the article/Supplementary Material. Further inquiries can be directed to the corresponding author.

## Supplementary Materials

The following supporting information can be downloaded at: https://www.mdpi.com/article/10.3390/molecules30091935/s1, Table S1. Tautomeric and transition states presented as relative energies (in kcal/mol units) in toluene (M06-2X/TZVP); Table S2. Dipole moments (in Debay units) in toluene (M06-2X/TZVP); Table S3. PESs presented as relative energies (in kcal/mol units) in acetonitrile (M06-2X/TZVP); Table S4. PESs presented as relative Gibbs energies (in kcal/mol units) in acetonitrile (M06-2X/TZVP); Figure S1. Ground-state energy diagrams of the studied compounds in toluene (M06-2X/TZVP); Figure S2. Dipole moments (in Debay units) of the tautomers and the transition states of selected compounds in toluene. Detailed information is available in Table S2; Figure S3. Dipole moments (in Debay units) of the tautomers and the transition states of selected compounds in toluene. Detailed information is available in Table S2; Figure S4. Simulated absorption spectra of the tautomers of **4CN** and **5NO2** in toluene.

## References

[1] Gilli G., Gilli P. (2013). The Nature of the Hydrogen Bond: Outline of a Comprehensive Hydrogen Bond Theory.

[2] Guerrero-Corella A., Fraile A., Alemán J. (2022). Intramolecular Hydrogen-Bond Activation: Strategies, Benefits, and Influence in Catalysis. ACS Org. Inorg. Au.

[3] Jabłoński M. (2021). Intramolecular Hydrogen Bonding 2021. Molecules.

[4] Baker E.N., Rossmann M.G., Arnold E. (2006). Hydrogen Bonding in Biological Macromolecules. International Tables for Crystallography.

[5] Jeffrey G.A., Saenger W. (1991). Hydrogen Bonding in Biological Structures.

[6] Pairas G.N., Tsoungas P.G. (2016). *H*-Bond: Τhe Chemistry-Biology *H*-Bridge. ChemistrySelect.

[7] Caron G., Kihlberg J., Ermondi G. (2019). Intramolecular Hydrogen Bonding: An Opportunity for Improved Design in Medicinal Chemistry. Med. Res. Rev..

[8] Kuhn B., Mohr P., Stahl M. (2010). Intramolecular Hydrogen Bonding in Medicinal Chemistry. J. Med. Chem..

[9] Hutchins K.M. (2018). Functional Materials Based on Molecules with Hydrogen-Bonding Ability: Applications to Drug Co-Crystals and Polymer Complexes. R. Soc. Open Sci..

[10] Wan Q., Thompson B.C. (2024). Control of Properties through Hydrogen Bonding Interactions in Conjugated Polymers. Adv. Sci..

[11] Deng Y., Zhang Q., Qu D.-H. (2023). Emerging Hydrogen-Bond Design for High-Performance Dynamic Polymeric Materials. ACS Mater. Lett..

[12] Shi X., Bao W. (2021). Hydrogen-Bonded Conjugated Materials and Their Application in Organic Field-Effect Transistors. Front. Chem..

[13] Hynes J.T., Klinman J.P., Limbach H.-H., Schowen R.L. (2006). Hydrogen-Transfer Reactions.

[14] Joshi H.C., Antonov L. (2021). Excited-State Intramolecular Proton Transfer: A Short Introductory Review. Molecules.

[15] Kwon J.E., Park S.Y. (2011). Advanced Organic Optoelectronic Materials: Harnessing Excited-State Intramolecular Proton Transfer (ESIPT) Process. Adv. Mater..

[16] Tsutsui M., Taniguchi M. (2012). Single Molecule Electronics and Devices. Sensors.

[17] Sakai K., Tsuzuki T., Itoh Y., Ichikawa M., Taniguchi Y. (2005). Using Proton-Transfer Laser Dyes for Organic Laser Diodes. Appl. Phys. Lett..

[18] Chen K.-Y., Hsieh C.-C., Cheng Y.-M., Lai C.-H., Chou P.-T. (2006). Extensive Spectral Tuning of the Proton Transfer Emission from 550 to 675 Nm via a Rational Derivatization of 10-Hydroxybenzo[h]Quinoline. Chem. Commun..

[19] Demchenko A.P., Tang K.-C., Chou P.-T. (2013). Excited-State Proton Coupled Charge Transfer Modulated by Molecular Structure and Media Polarization. Chem. Soc. Rev..

[20] Chen C.-L., Chen Y.-T., Demchenko A.P., Chou P.-T. (2018). Amino Proton Donors in Excited-State Intramolecular Proton-Transfer Reactions. Nat. Rev. Chem..

[21] Minkin V.I., Tsukanov A.V., Dubonosov A.D., Bren V.A. (2011). Tautomeric Schiff Bases: Iono-, Solvato-, Thermo- and Photochromism. J. Mol. Struct..

[22] Nikolaeva O.G., Popova O.S., Dubonosova I.V., Karlutova O.Y., Dubonosov A.D., Bren V.A., Minkin V.I. (2022). Spectral-Luminescent and Ionochromic Properties of Azomethine Imine-Coumarin Conjugates. Russ. J. Gen. Chem..

[23] Sedgwick A.C., Wu L., Han H.-H., Bull S.D., He X.-P., James T.D., Sessler J.L., Tang B.Z., Tian H., Yoon J. (2018). Excited-State Intramolecular Proton-Transfer (ESIPT) Based Fluorescence Sensors and Imaging Agents. Chem. Soc. Rev..

[24] Li Y., Dahal D., Abeywickrama C.S., Pang Y. (2021). Progress in Tuning Emission of the Excited-State Intramolecular Proton Transfer (ESIPT)-Based Fluorescent Probes. ACS Omega.

[25] Chen L., Fu P., Wang H., Pan M. (2021). Excited-State Intramolecular Proton Transfer (ESIPT) for Optical Sensing in Solid State. Adv. Opt. Mater..

[26] Shekhovtsov N.A., Vorob’eva S., Nikolaenkova E.B., Ryadun A.A., Krivopalov V.P., Gourlaouen C., Bushuev M.B. (2023). Complexes on the Base of a Proton Transfer Capable Pyrimidine Derivative: How Protonation and Deprotonation Switch Emission Mechanisms. Inorg. Chem..

[27] Shekhovtsov N.A., Nikolaenkova E.B., Ryadun A.A., Vorobyeva S.N., Krivopalov V.P., Bushuev M.B. (2023). Dual Emission of ESIPT-Capable 2-(2-Hydroxyphenyl)-4-(1 *H* -Pyrazol-1-Yl)Pyrimidines: Interplay of Fluorescence and Phosphorescence. New J. Chem..

[28] Fu P.-Y., Yi S.-Z., Pan M., Su C.-Y. (2023). Excited-State Intramolecular Proton Transfer (ESIPT) Based Metal–Organic Supramolecular Optical Materials: Energy Transfer Mechanism and Luminescence Regulation Strategy. Acc. Mater. Res..

[29] Shekhovtsov N.A., Ryadun A.A., Plyusnin V.F., Nikolaenkova E.B., Tikhonov A.Y., Bushuev M.B. (2022). First 1-Hydroxy-1 *H* -Imidazole-Based ESIPT Emitter with an O–H⋯O Intramolecular Hydrogen Bond: ESIPT-Triggered TICT and Speciation in Solution. New J. Chem..

[30] Shekhovtsov N.A., Nikolaenkova E.B., Berezin A.S., Plyusnin V.F., Vinogradova K.A., Naumov D.Y., Pervukhina N.V., Tikhonov A.Y., Bushuev M.B. (2022). Tuning ESIPT-Coupled Luminescence by Expanding π-Conjugation of a Proton Acceptor Moiety in ESIPT-Capable Zinc(II) Complexes with 1-Hydroxy-1*H*-Imidazole-Based Ligands. Dalton Trans..

[31] Trannoy V., Léaustic A., Gadan S., Guillot R., Allain C., Clavier G., Mazerat S., Geffroy B., Yu P. (2021). A Highly Efficient Solution and Solid State ESIPT Fluorophore and Its OLED Application. New J. Chem..

[32] Shekhovtsov N.A., Ryadun A.A., Bushuev M.B. (2021). Luminescence of a Zinc(II) Complex with a Protonated 1-Hydroxy-1*H*-imidazole ESIPT Ligand: Direct Excitation of a Tautomeric Form. ChemistrySelect.

[33] Shekhovtsov N.A., Nikolaenkova E.B., Berezin A.S., Plyusnin V.F., Vinogradova K.A., Naumov D.Y., Pervukhina N.V., Tikhonov A.Y., Bushuev M.B. (2021). A 1-Hydroxy-1*H*-imidazole ESIPT Emitter Demonstrating anti-Kasha Fluorescence and Direct Excitation of a Tautomeric Form. ChemPlusChem.

[34] Petdee S., Chaiwai C., Benchaphanthawee W., Nalaoh P., Kungwan N., Namuangruk S., Sudyoadsuk T., Promarak V. (2021). Imidazole-Based Solid-State Fluorophores with Combined ESIPT and AIE Features as Self-Absorption-Free Non-Doped Emitters for Electroluminescent Devices. Dye. Pigment..

[35] Long Y., Mamada M., Li C., Dos Santos P.L., Colella M., Danos A., Adachi C., Monkman A. (2020). Excited State Dynamics of Thermally Activated Delayed Fluorescence from an Excited State Intramolecular Proton Transfer System. J. Phys. Chem. Lett..

[36] Smith T.P., Zaklika K.A., Thakur K., Walker G.C., Tominaga K., Barbara P.F. (1992). Ultrafast Studies on Proton Transfer in Photostabilizers. J. Photochem. Photobiol. Chem..

[37] Ewing G.W., Steck E.A. (1946). Absorption Spectra of Heterocyclic Compounds. I. Quinolinols and Isoquinolinols ^1^. J. Am. Chem. Soc..

[38] Mason S.F. (1957). The Tautomerism of N-Heteroaromatic Hydroxy-Compounds. Part I. Infrared Spectra. J. Chem. Soc. Resumed.

[39] Mason S.F. (1957). The Tautomerism of N-Heteroaromatic Hydroxy-Compounds. Part II. Ultraviolet Spectra. J. Chem. Soc. Resumed.

[40] Mason S.F., Philp J., Smith B.E. (1968). Prototropic Equilibria of Electronically Excited Molecules. Part II. 3-, 6-, and 7-Hydroxyquinoline. J. Chem. Soc. Inorg. Phys. Theor..

[41] Lee S.-I., Jang D.-J. (1995). Proton Transfers of Aqueous 7-Hydroxyquinoline in the First Excited Singlet, Lowest Triplet, and Ground States. J. Phys. Chem..

[42] García-Ochoa I., Bisht P.B., Sánchez F., Martinez-Atáz E., Santos L., Tripathi H.B., Douhal A. (1998). Experimental and Theoretical Studies of the Proton-Hopping Reaction of 7-Hydroxyquinoline in Viscous Hydroxylic Media. J. Phys. Chem. A.

[43] Chou P.-T., Wei C.-Y., Chris Wang C.-R., Hung F.-T., Chang C.-P. (1999). Proton-Transfer Tautomerism of 7-Hydroxyquinolines Mediated by Hydrogen-Bonded Complexes. J. Phys. Chem. A.

[44] Kwon O.-H., Lee Y.-S., Yoo B.K., Jang D.-J. (2006). Excited-State Triple Proton Transfer of 7-Hydroxyquinoline along a Hydrogen-Bonded Alcohol Chain: Vibrationally Assisted Proton Tunneling. Angew. Chem. Int. Ed..

[45] Park S.-Y., Lee Y.-S., Kwon O.-H., Jang D.-J. (2009). Proton Transport of Water in Acid–Base Reactions of 7-Hydroxyquinoline. Chem. Commun..

[46] Park S.-Y., Jang D.-J. (2012). Excited-State Hydrogen Relay along a Blended-Alcohol Chain as a Model System of a Proton Wire: Deuterium Effect on the Reaction Dynamics. Phys. Chem. Chem. Phys..

[47] Kumpulainen T., Lang B., Rosspeintner A., Vauthey E. (2017). Ultrafast Elementary Photochemical Processes of Organic Molecules in Liquid Solution. Chem. Rev..

[48] Vetokhina V., Nowacki J., Pietrzak M., Rode M.F., Sobolewski A.L., Waluk J., Herbich J. (2013). 7-Hydroxyquinoline-8-Carbaldehydes. 1. Ground- and Excited-State Long-Range Prototropic Tautomerization. J. Phys. Chem. A.

[49] Fang W.-H. (1998). Ab Initio Study of the Triple-Proton-Transfer Reactions of Ground and Excited States of 7-Hydroxyquinoline in Methanol Solution. J. Am. Chem. Soc..

[50] Fang W.-H. (1999). Theoretical Characterization of the Structures and Reactivity of 7-Hydroxyquinoline−(H_2_O)*_n_*(*n* = 1−3) Complexes. J. Phys. Chem. A.

[51] Tanner C. (2003). Probing the Threshold to H Atom Transfer Along a Hydrogen-Bonded Ammonia Wire. Science.

[52] Fernández-Ramos A., Martínez-Núñez E., Vázquez S.A., Ríos M.A., Estévez C.M., Merchán M., Serrano-Andrés L. (2007). Hydrogen Transfer vs Proton Transfer in 7-Hydroxy-Quinoline·(NH_3_)_3_: A CASSCF/CASPT2 Study. J. Phys. Chem. A.

[53] Al-Lawatia N., Husband J., Steinbrecher T., Abou-Zied O.K. (2011). Tautomerism in 7-Hydroxyquinoline: A Combined Experimental and Theoretical Study in Water. J. Phys. Chem. A.

[54] Bekçioğlu G., Allolio C., Ekimova M., Nibbering E.T.J., Sebastiani D. (2014). Competition between Excited State Proton and OH^−^ Transport via a Short Water Wire: Solvent Effects Open the Gate. Phys. Chem. Chem. Phys..

[55] Kerdpol K., Daengngern R., Meeprasert J., Namuangruk S., Kungwan N. (2016). Theoretical Insights into Photoinduced Proton Transfer of 7-Hydroxyquinoline via Intermolecular Hydrogen-Bonded Wire of Mixed Methanol and Water. Theor. Chem. Acc..

[56] Fang H., Kim Y. (2017). Hydrogen-Bonded Channel-Dependent Mechanism of Long-Range Proton Transfer in the Excited-State Tautomerization of 7-Hydroxyquinoline: A Theoretical Study. Theor. Chem. Acc..

[57] Mori Y. (2018). Reaction Pathway and H/D Kinetic Isotope Effects of the Triple Proton Transfer in a 7-Hydroxyquinoline-Methanol Complex in the Ground State: A Computational Approach. J. Phys. Org. Chem..

[58] Miura M., Harada J., Ogawa K. (2007). Temperature-Induced Reversal of Proton Tautomerism: Role of Hydrogen Bonding and Aggregation in 7-Hydroxyquinolines. J. Phys. Chem. A.

[59] Sekine M., Nagai Y., Sekiya H., Nakata M. (2009). Photoinduced Hydrogen-Atom Eliminations of 6-Hydroxyquinoline and 7-Hydroxyquinoline Studied by Low-Temperature Matrix-Isolation Infrared Spectroscopy and Density-Functional-Theory Calculations. J. Phys. Chem. A.

[60] Nagai Y., Saita K., Sakota K., Nanbu S., Sekine M., Nakata M., Sekiya H. (2010). Electronic Spectra of Two Long-Lived Photoproducts: Double-Proton Transfer in 7-Hydroxyquinoline Dimer in a 2-Methyltetrahydrofuran Glass Matrix. J. Phys. Chem. A.

[61] Lim H., Park S.-Y., Jang D.-J. (2010). Excited-State Double Proton Transfer Dynamics of Model DNA Base Pairs: 7-Hydroxyquinoline Dimers. J. Phys. Chem. A.

[62] Sekine M., Nagai Y., Sekiya H., Nakata M. (2010). Electronic Absorption Spectra of Photoreaction Intermediates of 7-Hydroxyquinoline Monomer in a Low-Temperature Argon Matrix and Time-Dependent Density-Functional-Theory Calculations. Chem. Phys. Lett..

[63] Konijnenberg J., Ekelmans G.B., Huizer A.H., Varma C.A.G.O. (1989). Mechanism and Solvent Dependence of the Solvent-Catalysed Pseudo-Intramolecular Proton Transfer of 7-Hydroxyquinoline in the First Electronically Excited Singlet State and in the Ground State of Its Tautomer. J. Chem. Soc. Faraday Trans. 2.

[64] Tang K.-C., Chen C.-L., Chuang H.-H., Chen J.-L., Chen Y.-J., Lin Y.-C., Shen J.-Y., Hu W.-P., Chou P.-T. (2011). A Genuine Intramolecular Proton Relay System Undergoing Excited-State Double Proton Transfer Reaction. J. Phys. Chem. Lett..

[65] Kumar G., Paul K., Luxami V. (2020). Deciphering the Excited State Intramolecular Charge-Coupled Double Proton Transfer in an Asymmetric Quinoline–Benzimidazole System. New J. Chem..

[66] Jalink C.J., van Ingen W.M., Huizer A.H., Varma C.A.G.O. (1991). Prospects for Using Photoinduced Intramolecular Proton Transfer to Study the Dynamics of Conformational Changes in Flexible Molecular Chains. J. Chem. Soc. Faraday Trans..

[67] Jalink C.J., Huizer A.H., Varma C.A.G.O. (1992). Rate-Limiting Action of a Proton Crane in Long-Range Intramolecular Proton Transfer. J. Chem. Soc. Faraday Trans..

[68] Rehhagen C., Argüello Cordero M.A., Kamounah F.S., Deneva V., Angelov I., Krupp M., Svenningsen S.W., Pittelkow M., Lochbrunner S., Antonov L. (2024). Reversible Switching Based on Truly Intramolecular Long-Range Proton Transfer─Turning the Theoretical Concept into Experimental Reality. J. Am. Chem. Soc..

[69] van der Loop T.H., Ruesink F., Amirjalayer S., Sanders H.J., Buma W.J., Woutersen S. (2014). Unraveling the Mechanism of a Reversible Photoactivated Molecular Proton Crane. J. Phys. Chem. B.

[70] Slavova S., Antonov L. (2024). Theoretical Understanding of the Long-Range Proton Transfer Mechanism in 7-Hydroxy Quinoline-Based Molecular Switches: Varma’s Proton Crane and Its Analogues. J. Phys. Chem. A.

[71] Lapinski L., Nowak M.J., Nowacki J., Rode M.F., Sobolewski A.L. (2009). A Bistable Molecular Switch Driven by Photoinduced Hydrogen-Atom Transfer. ChemPhysChem.

[72] Rode M.F., Sobolewski A.L. (2010). Effect of Chemical Substituents on the Energetical Landscape of a Molecular Photoswitch: An Ab Initio Study. J. Phys. Chem. A.

[73] Csehi A., Illés L., Halász G.J., Vibók Á. (2013). The Effect of Chemical Substituents on the Functionality of a Molecular Switch System: A Theoretical Study of Several Quinoline Compounds. Phys. Chem. Chem. Phys..

[74] Csehi A., Halász G.J., Vibók Á. (2014). Molecular Switch Properties of 7-Hydroxyquinoline Compounds. Int. J. Quantum Chem..

[75] Slavova S., Antonov L. (2024). Azaindolizine Proton Cranes Attached to 7-Hydroxyquinoline and 3-Hydroxypyridine: A Comparative Theoretical Study. Phys. Chem. Chem. Phys..

[76] Zaharieva L., Nedeltcheva-Antonova D., Antonov L. (2024). 8-(Pyridin-2-Yl)Quinolin-7-Ol and Beyond: Theoretical Design of Tautomeric Molecular Switches with Pyridine as a Proton Crane Unit. Chemistry.

[77] Zaharieva L., Angelov I., Antonov L. (2024). Stationary External Electric Field—Mimicking the Solvent Effect on the Ground-State Tautomerism and Excited-State Proton Transfer in 8-(Benzo[d]Thiazol-2-Yl)Quinolin-7-Ol. Molecules.

[78] Georgiev A., Yordanov D., Ivanova N., Deneva V., Vassilev N., Kamounah F.S., Pittelkow M., Crochet A., Fromm K.M., Antonov L. (2021). 7-OH Quinoline Schiff Bases: Are They the Long Awaited Tautomeric Bistable Switches?. Dye. Pigment..

[79] Woolfe G.J., Melzig M., Schneider S., Dörr F. (1983). The Role of Tautomeric and Rotameric Species in the Photophysics of 2-(2′-Hydroxyphenyl)Benzoxazole. Chem. Phys..

[80] Barbatti M., Aquino A.J.A., Lischka H., Schriever C., Lochbrunner S., Riedle E. (2009). Ultrafast Internal Conversion Pathway and Mechanism in 2-(2′-Hydroxyphenyl)Benzothiazole: A Case Study for Excited-State Intramolecular Proton Transfer Systems. Phys. Chem. Chem. Phys..

[81] Lochbrunner S., Antonov L. (2013). Femtosecond Pump–Probe Spectroscopy of Photoinduced Tautomerism. Tautomerism.

[82] LeGourriérec D., Kharlanov V.A., Brown R.G., Rettig W. (2000). Excited-State Intramolecular Proton Transfer (ESIPT) in 2-(2′-Hydroxyphenyl)-Oxazole and -Thiazole. J. Photochem. Photobiol. Chem..

[83] Frisch M.J., Trucks G.W., Schlegel H.B., Scuseria G.E., Robb M.A., Cheeseman J.R., Scalmani G., Barone V., Petersson G.A., Nakatsuji H. (2016). Gaussian 16 Rev. C.01.

[84] Tomasi J., Mennucci B., Cammi R. (2005). Quantum Mechanical Continuum Solvation Models. Chem. Rev..

[85] Peng C., Ayala P.Y., Schlegel H.B., Frisch M.J. (1996). Using Redundant Internal Coordinates to Optimize Equilibrium Geometries and Transition States. J. Comput. Chem..

[86] Weinhold F., Landis C.R. (2005). Valency and Bonding: A Natural Bond Orbital Donor-Acceptor Perspective.

[87] Zhao Y., Truhlar D.G. (2008). Density Functionals with Broad Applicability in Chemistry. Acc. Chem. Res..

[88] Zhao Y., Truhlar D.G. (2008). The M06 Suite of Density Functionals for Main Group Thermochemistry, Thermochemical Kinetics, Noncovalent Interactions, Excited States, and Transition Elements: Two New Functionals and Systematic Testing of Four M06-Class Functionals and 12 Other Functionals. Theor. Chem. Acc..

[89] Weigend F., Ahlrichs R. (2005). Balanced Basis Sets of Split Valence, Triple Zeta Valence and Quadruple Zeta Valence Quality for H to Rn: Design and Assessment of Accuracy. Phys. Chem. Chem. Phys..

[90] Kawauchi S., Antonov L. (2013). Description of the Tautomerism in Some Azonaphthols. J. Phys. Org. Chem..

[91] Rayne S., Forest K. (2016). A Comparative Examination of Density Functional Performance against the ISOL24/11 Isomerization Energy Benchmark. Comput. Theor. Chem..

[92] Antonov L. (2019). Tautomerism in Azo and Azomethyne Dyes: When and If Theory Meets Experiment. Molecules.

[93] Deneva V., Vassilev N.G., Hristova S., Yordanov D., Hayashi Y., Kawauchi S., Fennel F., Völzer T., Lochbrunner S., Antonov L. (2020). Chercher de l’eau: The Switching Mechanism of the Rotary Switch Ethyl-2-(2-(Quinolin-8-Yl)Hydrazono)-2-(Pyridin-2-Yl)Acetate. Comput. Mater. Sci..

[94] Bauernschmitt R., Ahlrichs R. (1996). Treatment of Electronic Excitations within the Adiabatic Approximation of Time Dependent Density Functional Theory. Chem. Phys. Lett..

[95] Improta R., Barone V. (2011). UV-Visible Absorption and Emission Energies in Condensed Phase by PCM/TD-DFT Methods. Computational Strategies for Spectroscopy.

[96] Liu J., Liang W. (2011). Analytical Approach for the Excited-State Hessian in Time-Dependent Density Functional Theory: Formalism, Implementation, and Performance. J. Chem. Phys..

[97] Yanai T., Tew D.P., Handy N.C. (2004). A New Hybrid Exchange–Correlation Functional Using the Coulomb-Attenuating Method (CAM-B3LYP). Chem. Phys. Lett..

[98] Li R., Zheng J., Truhlar D.G. (2010). Density Functional Approximations for Charge Transfer Excitations with Intermediate Spatial Overlap. Phys. Chem. Chem. Phys..

[99] Wang J., Durbeej B. (2020). How Accurate Are TD-DFT Excited-state Geometries Compared to DFT Ground-state Geometries?. J. Comput. Chem..

[100] Mahato B., Panda A.N. (2021). Assessing the Performance of DFT Functionals for Excited-State Properties of Pyridine-Thiophene Oligomers. J. Phys. Chem. A.

[101] Sarkar R., Boggio-Pasqua M., Loos P.-F., Jacquemin D. (2021). Benchmarking TD-DFT and Wave Function Methods for Oscillator Strengths and Excited-State Dipole Moments. J. Chem. Theory Comput..

[102] Louant O., Champagne B., Liégeois V. (2018). Investigation of the Electronic Excited-State Equilibrium Geometries of Three Molecules Undergoing ESIPT: A RI-CC2 and TDDFT Study. J. Phys. Chem. A.

[103] Rode M.F., Nedeltcheva-Antonova D., Antonov L. (2022). Long-Range Proton Transfer in 7-Hydroxy-Quinoline-Based Azomethine Dyes: A Hidden Reason for the Low Efficiency. Molecules.

[104] Guo Y., Riplinger C., Becker U., Liakos D.G., Minenkov Y., Cavallo L., Neese F. (2018). Communication: An Improved Linear Scaling Perturbative Triples Correction for the Domain Based Local Pair-Natural Orbital Based Singles and Doubles Coupled Cluster Method [DLPNO-CCSD(T)]. J. Chem. Phys..

[105] Berraud-Pache R., Neese F., Bistoni G., Izsák R. (2020). Unveiling the Photophysical Properties of Boron-Dipyrromethene Dyes Using a New Accurate Excited State Coupled Cluster Method. J. Chem. Theory Comput..

[106] Becke A.D. (1993). Density-functional Thermochemistry. III. The Role of Exact Exchange. J. Chem. Phys..

[107] Nedeltcheva-Antonova D., Antonov L. (2024). Ground-State Tautomerism and Excited-State Proton Transfer in 7-Hydroxy-4-Methyl-8-((Phenylimino)Methyl)-2H-Chromen-2-One as a Potential Proton Crane. Physchem.

