# Supplementary material for: Improving the Long-Range Intramolecular Proton Transfer—Further Molecular Design of the Successful Molecular Switch 8-(Benzo[d]thiazol-2-yl)quinolin-7-ol (HQBT)
Source: Molecules. 2025 Apr 26;30(9):1935. doi: 10.3390/molecules30091935 (PMC12073794; doi:10.3390/molecules30091935)
Supplement: Supplementary file 1 [file molecules-30-01935-s001.zip › molecules-3579628-supplementary.pdf]

Supplementary Materials

Improving the long-range intramolecular proton transfer – further molecular design of the successful molecular switch 8-(benzo[d]thiazol-2-yl)quinolin-7-ol (HQBT)

Daniela Nedeltcheva-Antonova [a,b] and Liudmil Antonov [a,*]

a) Institute of Electronics, Bulgarian Academy of Sciences, Sofia 1784, Bulgaria

b) Institute of Organic Chemistry with Centre of Phytochemistry, Bulgarian Academy of Sciences, Sofia 1113, Bulgaria

Table S1. Tautomeric and transition states presented as relative energies (in kcal/mol units) in toluene (M06-2X/TZVP).

	E	TS(E-KE)	KE	TS(KE-KK)	KK	TS(KK-K)	K
**HQBT**	0.00	5.99	5.47	30.50	6.18	10.52	6.45
**4F**	0.00	7.01	6.66	32.47	6.81	10.32	5.45
**4CN**	0.00	7.76	7.51	34.72	6.73	9.41	3.76
**5F**	0.00	6.37	5.94	31.89	6.53	10.53	6.09
**5CF3**	0.00	6.86	6.49	33.19	6.95	10.69	5.76
**5CN**	0.00	7.03	6.68	33.94	7.09	10.72	5.56
**5NO2**	0.00	7.24	6.91	34.53	7.21	10.66	5.32
**5NMe2**	0.00	5.33	4.65	28.49	5.49	10.34	6.83
**5NHMe2**	0.00			39.83	9.12	11.93	5.28
**6F**	0.00	6.57	6.18	31.86	6.92	10.94	6.37
**6CN**	0.00	6.71	6.30	33.96	6.87	10.60	5.70
**6NO2**	0.00	6.81	6.40	34.65	6.94	10.55	5.51
**7F**	0.00	6.44	5.99	32.42	6.76	10.82	6.27
**7CN**	0.00	7.00	6.62	34.29	7.62	11.37	6.31

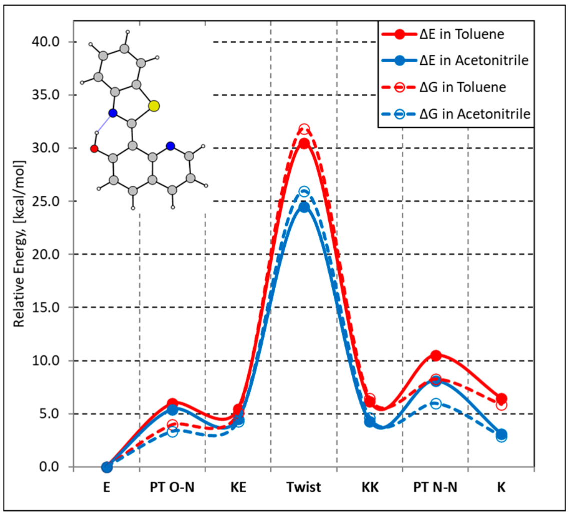
Relative Energy, [kcal/mol]
40.0
35.0
30.0
25.0
20.0
15.0
10.0
5.0
0.0
E
PT O-N
KE
Twist
KK
PT N-N
K
ΔE in Toluene
ΔE in Acetonitrile
ΔG in Toluene
ΔG in Acetonitrile

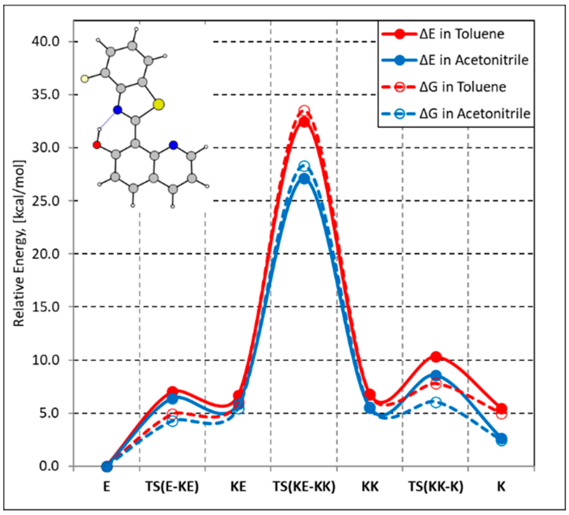
Relative Energy, [kcal/mol]
40.0
35.0
30.0
25.0
20.0
15.0
10.0
5.0
0.0
E
TS(E-KE)
KE
TS(KE-KK)
KK
TS(KK-K)
K
ΔE in Toluene
ΔE in Acetonitrile
ΔG in Toluene
ΔG in Acetonitrile

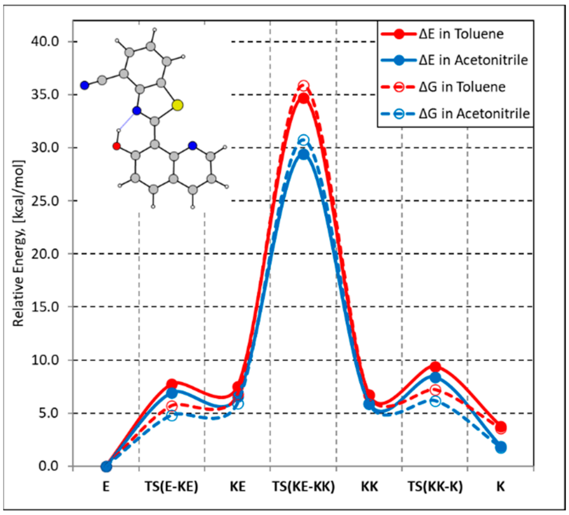
Relative Energy, [kcal/mol]
40.0
35.0
30.0
25.0
20.0
15.0
10.0
5.0
0.0
E
TS(E-KE)
KE
TS(KE-KK)
KK
TS(KK-K)
K
ΔE in Toluene
ΔE in Acetonitrile
ΔG in Toluene
ΔG in Acetonitrile

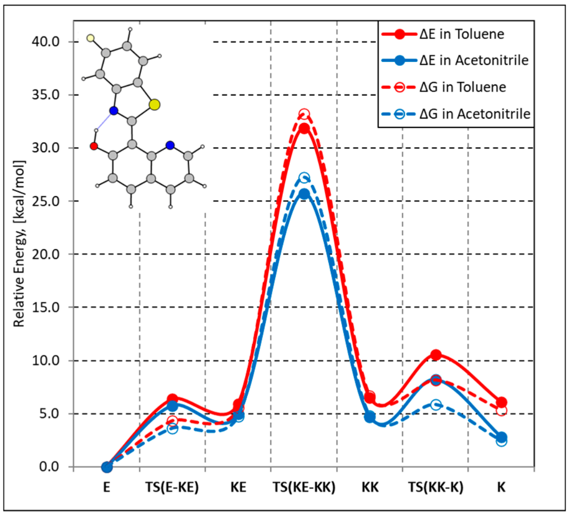
Relative Energy, [kcal/mol]
40.0
35.0
30.0
25.0
20.0
15.0
10.0
5.0
0.0
E
TS(E-KE)
KE
TS(KE-KK)
KK
TS(KK-K)
K
ΔE in Toluene
ΔE in Acetonitrile
ΔG in Toluene
ΔG in Acetonitrile

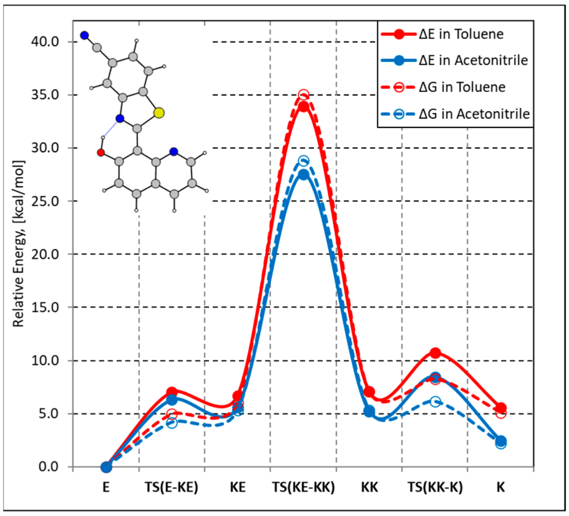
Relative Energy, [kcal/mol]
40.0
35.0
30.0
25.0
20.0
15.0
10.0
5.0
0.0
E
TS(E-KE)
KE
TS(KE-KK)
KK
TS(KK-K)
K
ΔE in Toluene
ΔE in Acetonitrile
ΔG in Toluene
ΔG in Acetonitrile

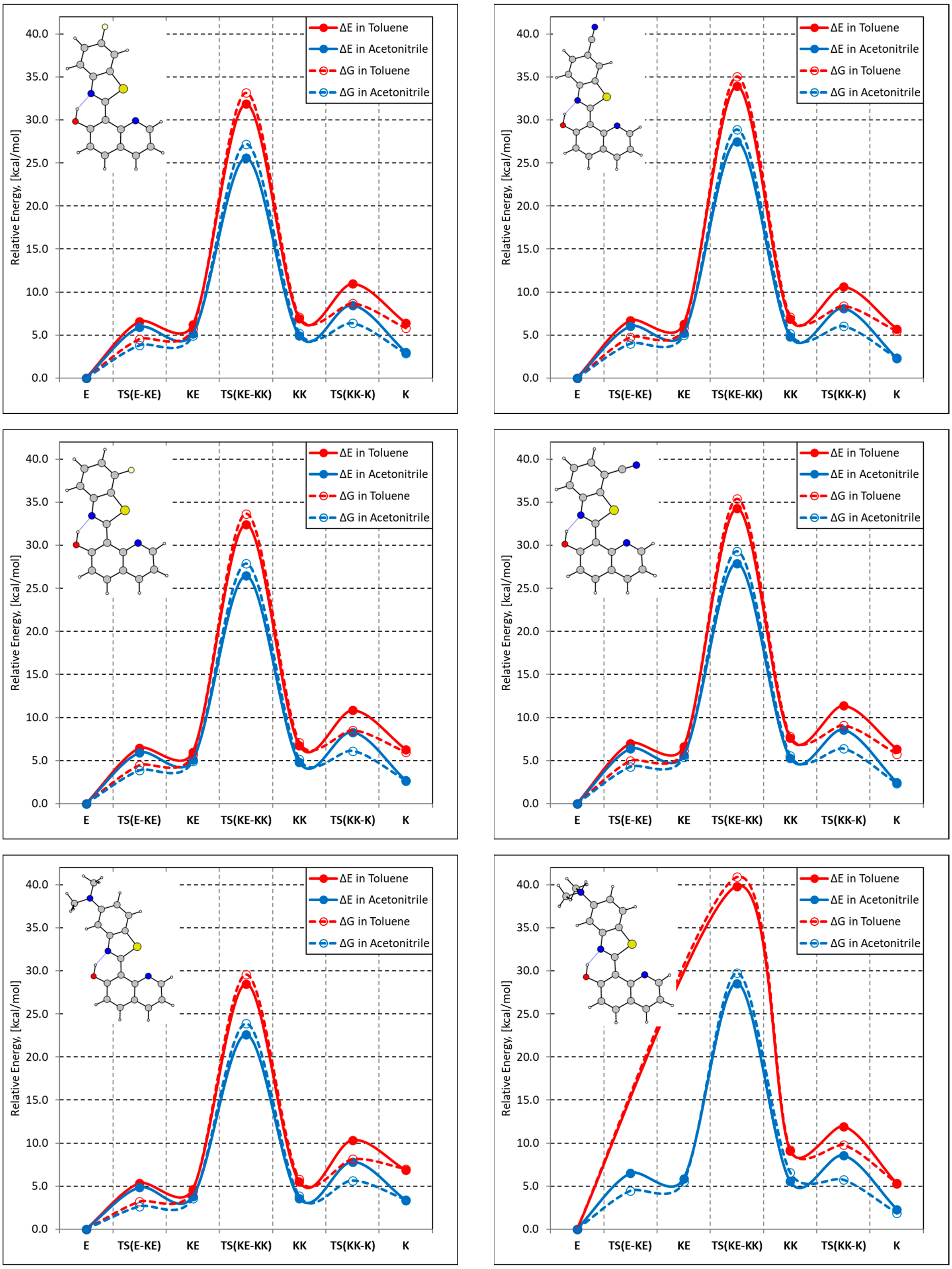
ΔE in Toluene
ΔE in Acetonitrile
ΔG in Toluene
ΔG in Acetonitrile
Relative Energy, [kcal/mol]
0.0
5.0
10.0
15.0
20.0
25.0
30.0
35.0
40.0
E
TS(E-KE)
KE
TS(KE-KK)
KK
TS(KK-K)
K

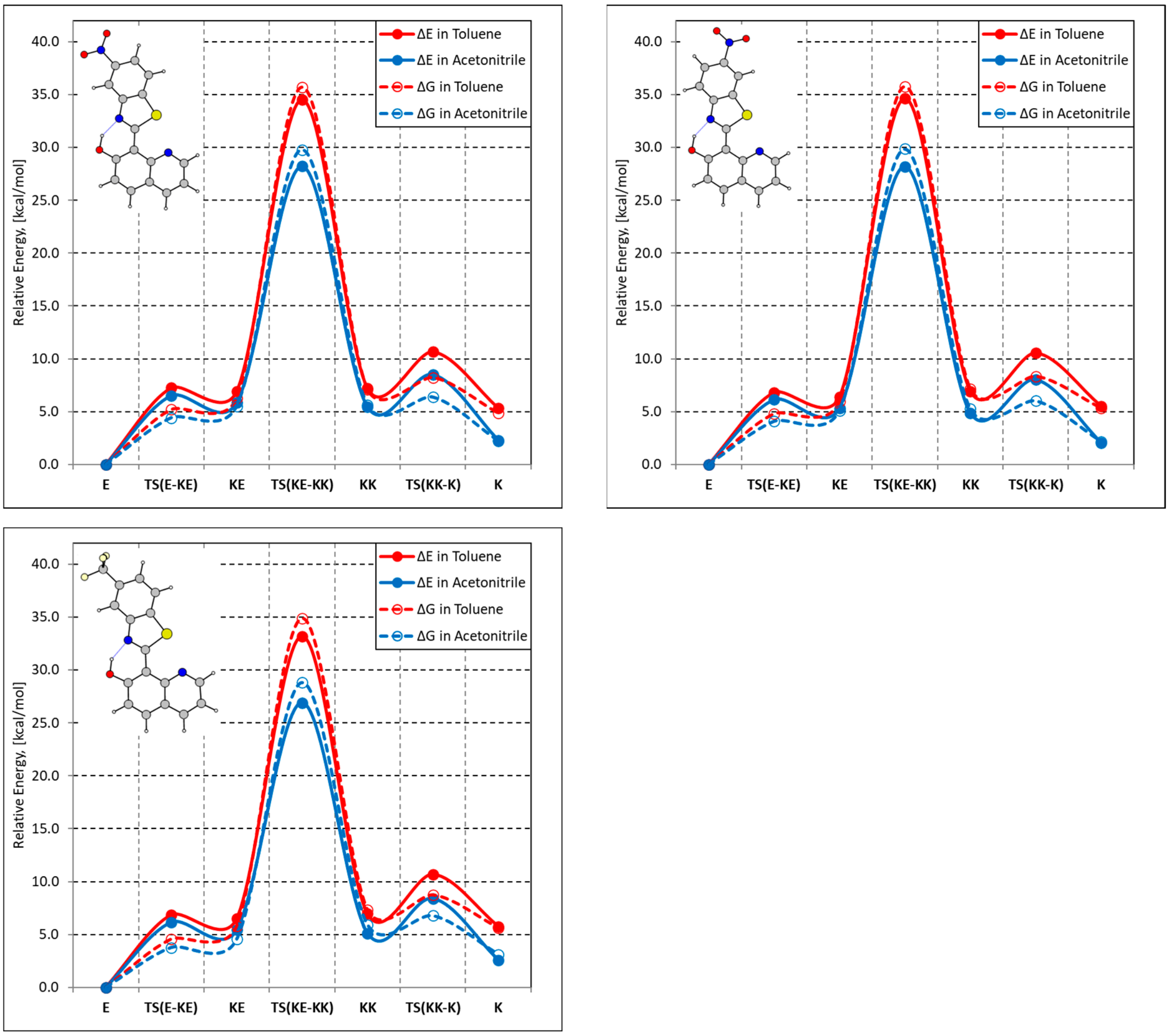

Figure S1. Ground-state energy diagrams of the studied compounds in toluene (M06-2X/TZVP).

Table S2. Dipole moments (in Debay units) in toluene (M06-2X/TZVP).

	**E**	**TS(E-KE)**	**KE**	**TS(KE-KK)**	**KK**	**TS(KK-K)**	**K**
**HQBT**	2.85	2.60	2.93	8.30	5.94	7.33	8.81
**4F**	4.46	4.36	4.54	8.07	4.23	5.55	7.33
**4CN**	7.66	7.53	7.62	8.90	1.33	2.32	4.59
**5F**	5.09	4.10	4.00	6.76	4.60	6.34	8.52
**5CF3**	6.93	5.58	5.34	5.66	4.40	6.28	9.01
**5CN**	8.99	7.55	7.27	5.01	4.67	6.47	9.54
**5NO2**	9.39	7.92	7.65	4.94	4.95	6.68	9.83
**5NMe2**	1.11	3.12	4.05	11.25	8.24	8.95	9.52
**6F**	4.16	2.48	2.23	6.90	6.50	8.21	10.27
**6CN**	8.03	5.87	5.26	5.43	9.05	10.99	13.70
**6NO2**	9.39	7.92	7.65	4.94	4.95	6.68	9.83
**7F**	2.81	1.02	1.01	8.18	7.55	9.12	10.87
**7CN**	4.61	2.65	2.23	9.27	10.64	12.32	14.29

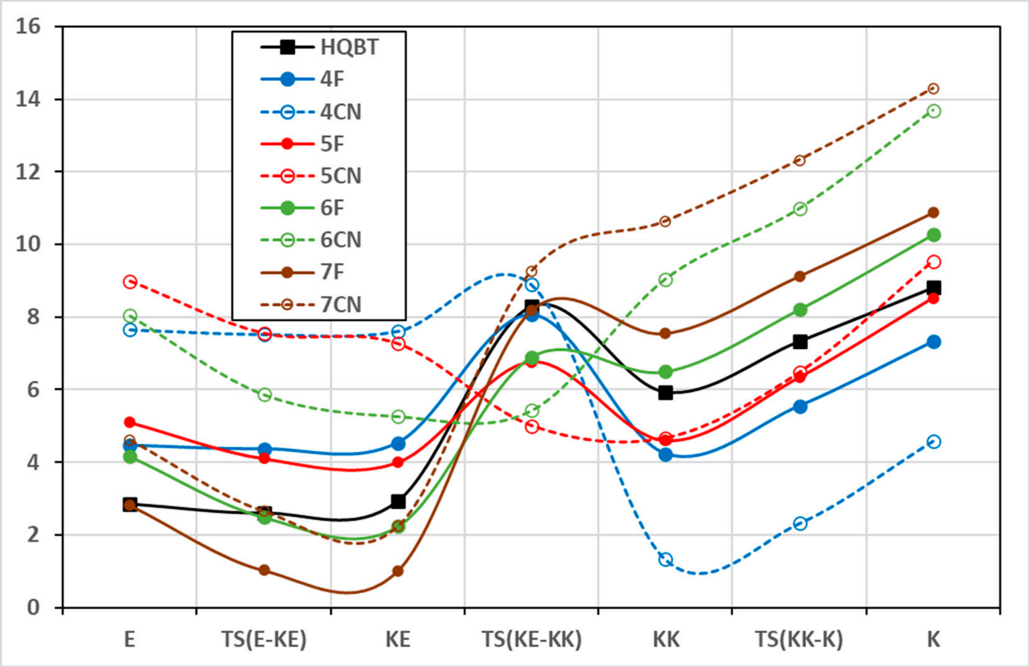

Figure S2. Dipole moments (in Debay units) of the tautomers and the transition states of selected compounds in toluene. Detailed information is available in Table S2.

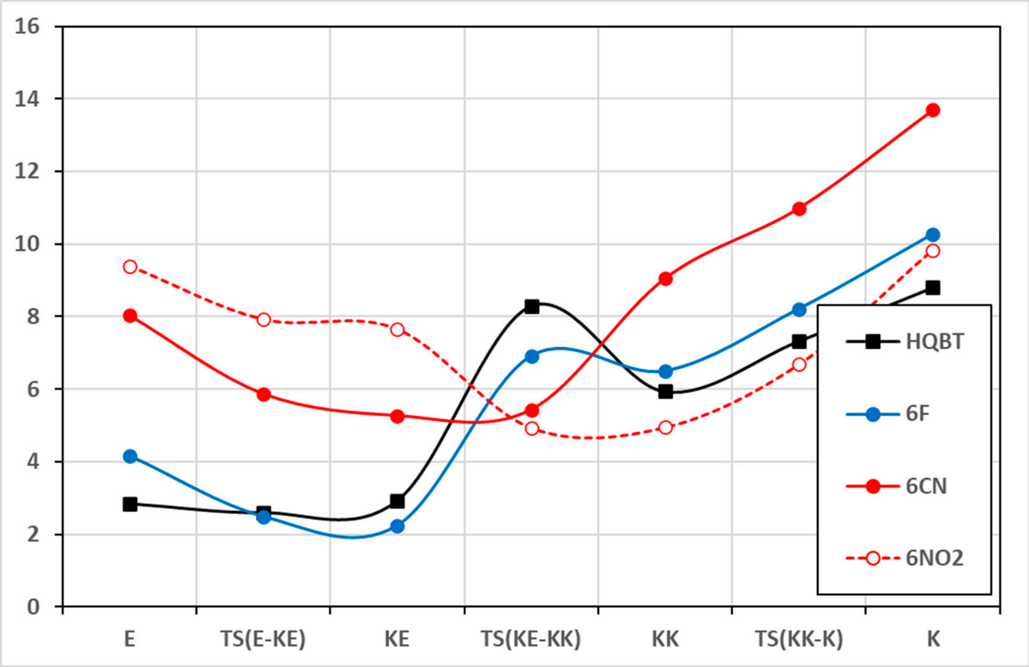

Figure S3. Dipole moments (in Debay units) of the tautomers and the transition states of selected compounds in toluene. Detailed information is available in Table S2.

Table S3. PESs presented as relative energies (in kcal/mol units) in acetonitrile (M06-2X/TZVP).

	E	TS(E-KE)	KE	TS(KE-KK)	KK	TS(KK-K)	K
**HQBT**	0.00	5.41	4.53	24.45	4.29	8.11	3.09
**4F**	0.00	6.40	5.86	27.13	5.54	8.57	2.63
**4CN**	0.00	6.93	6.56	29.40	5.94	8.40	1.86
**5F**	0.00	5.76	4.97	25.73	4.70	8.20	2.78
**5CF3**	0.00	6.15	5.45	26.90	5.11	8.40	2.57
**5CN**	0.00	6.30	5.60	27.54	5.24	8.46	2.43
**5NO2**	0.00	6.51	5.84	28.22	5.44	8.51	2.28
**5NMe2**	0.00	4.84	3.78	22.61	3.57	7.82	3.34
**5NHMe2**	0.00	6.49	5.88	28.56	5.54	8.55	2.29
**6F**	0.00	5.90	5.16	25.61	4.91	8.46	2.97
**6CN**	0.00	6.01	5.23	27.49	4.82	8.09	2.29
**6NO2**	0.00	6.13	5.35	28.21	4.88	8.03	2.04
**7F**	0.00	5.91	5.11	26.45	4.77	8.29	2.65
**7CN**	0.00	6.37	5.62	27.90	5.30	8.55	2.44

Table S4. PESs presented as relative Gibbs energies (in kcal/mol units) in acetonitrile (M06-2X/TZVP).

	E	TS(E-KE)	KE	TS(KE-KK)	KK	TS(KK-K)	K
**HQBT**	0.00	3.34	4.29	25.97	4.60	5.97	2.89
**4F**	0.00	4.26	5.45	28.31	5.52	6.06	2.45
**4CN**	0.00	4.81	5.93	30.76	5.88	6.18	1.77
**5F**	0.00	3.63	4.76	27.26	4.80	5.85	2.44
**5CF3**	0.00	3.77	4.54	28.81	5.92	6.77	3.12
**5CN**	0.00	4.18	5.32	28.84	5.33	6.15	2.24
**5NO2**	0.00	4.41	5.51	29.77	5.62	6.36	2.21
**5NMe2**	0.00	2.64	3.58	23.87	3.84	5.64	3.41
**5NHMe2**	0.00	4.45	5.51	29.75	6.58	5.74	1.85
**6F**	0.00	3.79	4.90	27.20	5.24	6.36	2.84
**6CN**	0.00	3.99	4.99	28.92	5.15	6.02	2.32
**6NO2**	0.00	4.08	5.11	29.86	5.24	6.01	2.16
**7F**	0.00	3.86	4.95	27.90	5.16	6.12	2.69
**7CN**	0.00	4.26	5.40	29.29	5.54	6.37	2.33

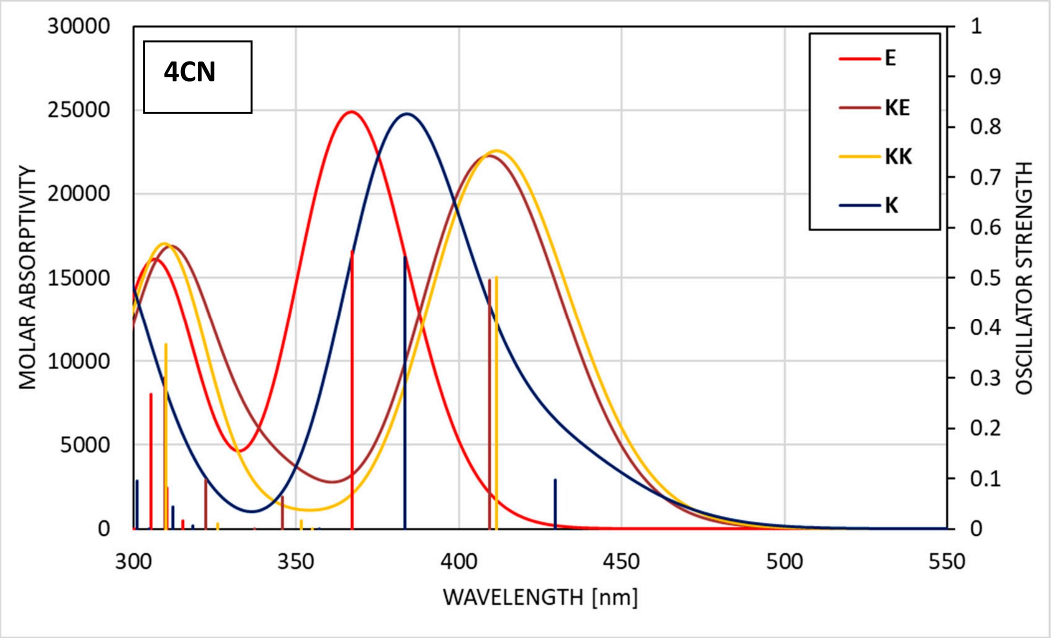

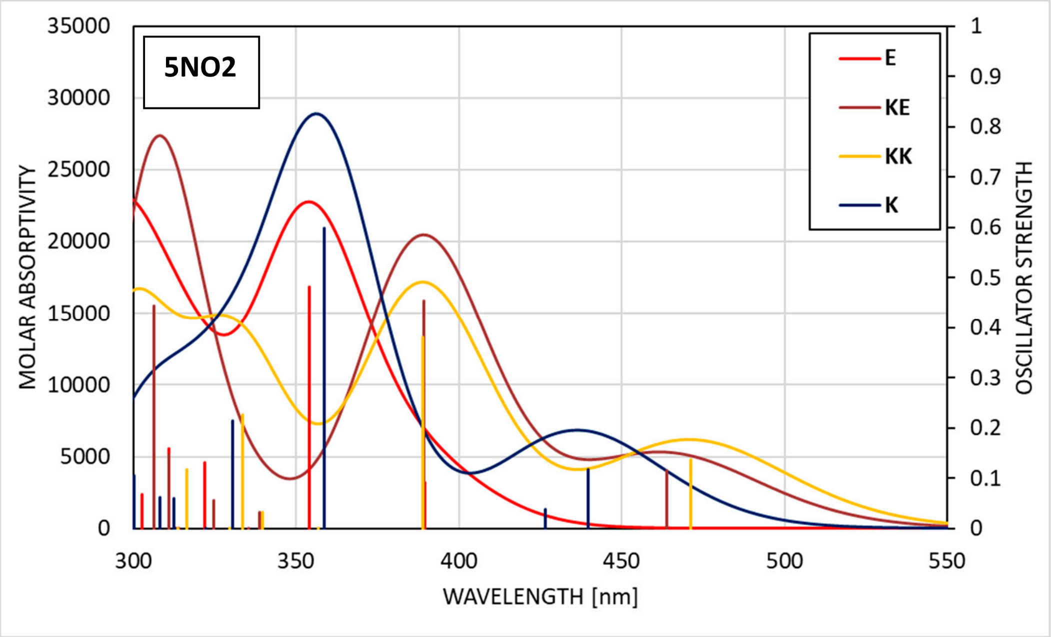

Figure S4. Simulated absorption spectra of the tautomers of **4CN** and **5NO2** in toluene.
